# Coralline algal structure is more sensitive to rate, rather than the magnitude, of ocean acidification

**DOI:** 10.1111/gcb.12351

**Published:** 2013-10-08

**Authors:** Nicholas A Kamenos, Heidi L Burdett, Elena Aloisio, Helen S Findlay, Sophie Martin, Charlotte Longbone, Jonathan Dunn, Stephen Widdicombe, Piero Calosi

**Affiliations:** * School of Geographical and Earth Sciences, University of GlasgowGlasgow, G12 8QQ, UK; † Department of Earth Sciences, University of St AndrewsFife, KY16 9AJ, UK; ‡ Marine Biology and Ecology Research Centre, School of Marine Science and Engineering, Plymouth UniversityDrake Circus, Plymouth, PL4 8AA, UK; § Plymouth Marine LaboratoryProspect Place, Plymouth, PL1 3DH, UK; ¶ CNRS, Laboratoire Adaptation et Diverstié en Milieu Marin, Station Biologique de RoscoffPlace Georges Teissier, 29688 Roscoff Cedex, France; ** Université Pierre et Marie Curie - Paris 6, Laboratoire Adaptation et Diversité en Milieu Marin, Station Biologique de RoscoffPlace Georges Teissier, 29688 Roscoff Cedex, France

**Keywords:** calcification, coralline algae, crustose coralline algae (CCA), maerl, ocean acidification, photosynthesis, raman, rate, respiration, rhodolith

## Abstract

Marine *p*CO_2_ enrichment *via* ocean acidification (OA), upwelling and release from carbon capture and storage (CCS) facilities is projected to have devastating impacts on marine biomineralisers and the services they provide. However, empirical studies using stable endpoint *p*CO_2_ concentrations find species exhibit variable biological and geochemical responses rather than the expected negative patterns. In addition, the carbonate chemistry of many marine systems is now being observed to be more variable than previously thought. To underpin more robust projections of future OA impacts on marine biomineralisers and their role in ecosystem service provision, we investigate coralline algal responses to realistically variable scenarios of marine *p*CO_2_ enrichment. Coralline algae are important in ecosystem function; providing habitats and nursery areas, hosting high biodiversity, stabilizing reef structures and contributing to the carbon cycle. Red coralline marine algae were exposed for 80 days to one of three pH treatments: (i) current pH (control); (ii) low pH (7.7) representing OA change; and (iii) an abrupt drop to low pH (7.7) representing the higher rates of pH change observed at natural vent systems, in areas of upwelling and during CCS releases. We demonstrate that red coralline algae respond differently to the rate and the magnitude of pH change induced by *p*CO_2_ enrichment. At low pH, coralline algae survived by increasing their calcification rates. However, when the change to low pH occurred at a fast rate we detected, using Raman spectroscopy, weaknesses in the calcite skeleton, with evidence of dissolution and molecular positional disorder. This suggests that, while coralline algae will continue to calcify, they may be structurally weakened, putting at risk the ecosystem services they provide. Notwithstanding evolutionary adaptation, the ability of coralline algae to cope with OA may thus be determined primarily by the rate, rather than magnitude, at which *p*CO_2_ enrichment occurs.

## Introduction

Marine *p*CO_2_ enrichment may occur *via* ocean acidification (OA) (Caldeira & Wickett, 2003), releases from natural CO_2_ vents (Hall-Spencer *et al*., 2008), upwelling (Feely *et al*., 2008), and sudden releases from carbon capture and storage (CCS) facilities (Blackford *et al*., 2009). Natural CO_2_ enrichment along with simulated OA and CCS leakages are used to investigate the responses of marine biomineralisers to projected OA. However, enrichment occurs at different rates with differing longevities in each release process.

Ocean acidification is projected to occur slowly over centennial time scales causing oceanic pH to drop 0.3–0.5 units by the end of this century (Caldeira & Wickett, 2003). CO_2_ releases from marine vent sites (Kroeker *et al*., 2012), biota (Anthony *et al*., 2012) and upwelling areas (Gruber *et al*., 2012) naturally alter marine *p*CO_2_ but are highly variable even at diel time scales (Hall-Spencer *et al*., 2008, Kroeker *et al*., 2012). Sudden CO_2_ leakage from CCS infrastructure (CO_2_ pipelines and underground geological storage reservoirs) into the overlying sea water may occur at even faster time scales (hours), accompanied by a sudden fall in pH (Blackford *et al*., 2009) with changes in pH being most acute at small spatial scales (meters) (Agnew & Taylor, 1986). Thus, while *p*CO_2_ enrichment will expose marine biomineralisers to reduced pH, the rates of exposure will vary significantly. Such variability is expected to be characteristic of coastal *p*CO_2_ enrichment over the coming century, unlike pelagic regions which are expected to remain more stable (Duarte *et al*., 2013). Therefore the rate, rather than the magnitude, of exposure may be critical in determining the ability of marine biomineralisers to cope with projected changes in carbonate chemistry and synchronous multiple stressors such as temperature. This may partially explain why organisms show variable responses to contemporary *p*CO_2_ enrichment of similar magnitude (Burdett *et al*., 2012, Melzner *et al*., 2009, Ries, 2011).

**Figure 1:**
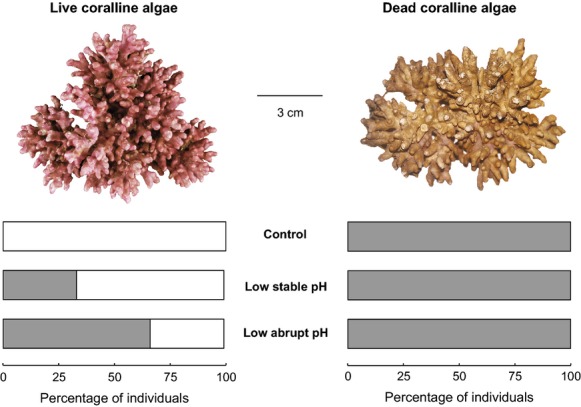
*Lithothamnion glaciale* thalli containing HCO_3_^−^. Percentage of *L. glaciale* thalli (live and dead) where HCO_3_^−^ was absent (white bars) or present (grey bars) after incubation for 80 days in control, low, stable pH, and low, abrupt pH treatments.

Red coralline algae (Fig. 1) have an established record as model biomineralisers for exploring the impact of high CO_2_ on marine calcifying biota (Burdett *et al*., 2012, Kroeker *et al*., 2012, Martin & Gattuso, 2009, Ragazzola *et al*., 2012, Ries, 2011). This is because (i) they use dissolved inorganic carbon to calcify and during photosynthesis (Martin & Gattuso, 2009), providing an excellent contrast to animal models; (ii) they are important in ecosystem function (e.g., Foster, 2001, Kamenos *et al*., 2004a, Kamenos *et al*., 2004b, Nelson, 2009); (iii) play a significant role in carbon cycling and reef stabilization (Nelson, 2009); and (iv) are utilised in ultra-high resolution palaeoenvironmental reconstructions (Burdett *et al*., 2011, Kamenos, 2010, Kamenos *et al*., 2012, Williams *et al*., 2011).

Coralline algae have a high-Mg skeleton (7.7–28.8 mol% MgCO_3_) (Chave, 1954, Kamenos *et al*., 2009), which is expected to make them particularly sensitive to marine *p*CO_2_ enrichment as high-Mg calcite is the most soluble form of calcium carbonate (Martin & Gattuso, 2009). However, algal responses to *p*CO_2_ enrichment do not show expected regular patterns. Some studies show negative patterns: for example, exposure to high *p*CO_2_ conditions inhibits coralline algal settlement (Kuffner *et al*., 2007), leads to coralline algal dissolution (Hall-Spencer *et al*., 2008, Martin & Gattuso, 2009), surface lesions (Martin & Gattuso, 2009), epithelial cell damage (Burdett *et al*., 2012) and modelled structural stress (Ragazzola *et al*., 2012). Conversely, other studies observe non-negative responses: for example, intracellular concentrations of the algal antioxidant dimethylsulphoniopropionate (DMSP) did not increase under gradual change to low pH (Burdett *et al*., 2012), OA induced higher coralline algal calcification (Martin *et al*., 2013) and did not cause coralline algae to change the CaCO_3_ polymorph deposited (a high Mg-calcite structure was maintained despite the higher stability of calcite or aragonite, Ries, 2011).

This study determines the effect of the rate and magnitude of pH change on the molecular structural integrity, calcification and respiration of living and dead coralline algae. It was hypothesised that, under faster rates of pH change, red coralline algae would exhibit the greatest response away from background levels; they are less likely to show beneficial phenotypic plastic responses to faster, compared to slower environmental change. Additionally, live red coralline algae were expected to be less impacted than dead red coralline algae due to the environmental buffering provided by the live epithelium.

## Materials and methods

### Experimental setup

Live and dead *Lithothamnion glaciale* thalli were hand collected from Loch Sween, Scotland (56°01.99′ N, 05°36.13′ W), in November 2009 using SCUBA from a depth of 7 m (thalli size: 4–6 cm diam.). *In situ*, the algae experience an annual temperature range of 5–16 °C, light levels of 30–120 μmol photons m^−2^ s^−1^ photosynthetically active radiation (PAR) and pH 8.1 (Rix *et al*., 2012). *Lithothamnion glaciale* was identified as described in Irvine and Chamberlain (1994). Thalli were maintained in aerated sea water at ca. 10 °C during transportation to Plymouth Marine Laboratory (Plymouth, UK) and were transferred to a 3000 l flow-through seawater system within one day of collection. Approximately 400 g of *L. glaciale* were separated into 24 experimental mesocosms (6 l volume, 28 × 19 × 16 cm): 12 containing live algae and 12 containing dead algae. Thalli were maintained in the system for one week (water temperature 11.63 ± 0.32 °C, salinity 34.9 ± 0.36 [mean ± SD]) prior the start of the ramping period to allow the thalli to recover from collection and adjust to laboratory conditions.

Coralline algae were incubated in three pH treatments for 80 days using the method developed by Findlay et al. (2008):

1. Control (pH 8.1, *n* = 12 [six with live algae and six with dead algae])
2. Low, stable pH change (pH 7.7, *n* = 6 [three with live algae and three with dead algae], rate of change from ambient: 0.05 pH units d^−1^ over 10 days prior to the experimental period) representing the A1FI IPCC year 2100 scenario (IPCC Core Writing Team Pachauri Rk & Reisinger a Eds, 2007).
3. Low, abrupt pH change (pH 8.1 to 7.7, *n* = 6 [three with live algae and three with dead algae], rate of change from ambient: 0.25 pH units d^−1^over two days beginning on day 52. The low, abrupt pH treatment represented a sudden reduction in pH following an acute injection of CO_2_ associated with CCS leaks, natural CO_2_ vent systems or areas of upwelling).

Rate of pH change in the low, abrupt pH treatment was significantly greater than in the low, stable pH treatment over the 2 days of change beginning on day 52 (F_1,43_ = 13.51, *P* = 0.001; multiple linear regression, assumptions met). Reduced pH levels were achieved by gradually increasing the bubbling of CO_2_ in the mesocosms.

### Carbonate system measurements

Seawater temperature, salinity (WTW LF187 combination temperature and salinity probe), pH_NBS_ (Metrohm, 826 pH mobile with a Metrohm glass electrode) and dissolved oxygen (1302 Oxygen Electrode; Strathkelvin Instruments, Glasgow, UK) were monitored daily. Nutrient concentrations (nitrate, nitrite, phosphate, silicate and ammonium) and total alkalinity (A_T_) were monitored weekly to ensure seawater quality was maintained (Table 1 and Table S1). Mesocosms were maintained at ambient temperature (11.63 ± 0.32 °C, mean ± SD) and light (90 μmol photons m^−2^ s^−1^; ca. 10 h light: 14 h dark). Nutrients were analysed with an autoanalyser (Bran+Luebbe, Norderstedt, Germany) using standard methods (Brewer & Riley, 1965, Grasshoff, 1976, Kirkwood, 1989, Mantoura & Woodward, 1983, Zhang & Chi, 2002). Total alkalinity was measured by poisoning according to Dickson et al. (2007) then analysing via potentiometric titration using an Apollo SciTech Alkalinity Titrator Model AS-ALK2 (Apollo SciTech, Bogart, GA, USA) and Batch 100 certified reference materials from Andrew Dickson.

**Table 1:** Mean ± SD experimental system values over the 80 days experimental period for measured temperature (*n* = 80), salinity (*n* = 80), pH (*n* = 80) and alkalinity (*n* = 12) and calculated, dissolved inorganic carbon (DIC), bicarbonate (HCO_3_^−^), carbonate (CO_3_^2−^), *p*CO_2_, calcite saturation state (Ω_cal_) and aragonite saturation state (Ω_arag_) for the three pH treatments.

	Control	Low, stable pH	Low, abrupt pH
Temperature (°C)	11.74 ± 0.34	11.51 ± 0.28	11.64 ± 0.33
Salinity	34.9 ± 0.34	35.0 ± 0.32	34.9 ± 0.41
pH (T)	8.18 ± 0.10	7.70 ± 0.14	7.75 ± 0.40
Alkalinity (μmol kg^−1^)	2975 ± 443	2964 ± 467	2991 ± 414
DIC (μmol kg^−1^)	2717 ± 420	2850 ± 489	3023 ± 556
HCO_3_^−^ (μmol kg^−1^)	2498 ± 395	2696 ± 475	2810 ± 463
CO_3_^2−^(μmol kg^−1^)	198 (±53)	110 (±26)	73 (±40)
*p*CO_2_	498 ± 161	1081 ± 488	2778 ± 4047
Ω_cal_	4.7 ± 1.26	2.62 ± 0.61	1.74 ± 0.94
Ω_arag_	3.0 ± 0.81	1.67 ± 0.39	1.11 ± 0.60

### Carbonate system calculations

Measured pH and A_T_ data were used to calculate carbonate and bicarbonate ion concentrations and calcite and aragonite (Ω_cal_ and Ω_arag_ respectively) saturation states using CO2SYS (Pierrot *et al*., 2006) with dissociation constants from Mehrbach et al. (1973) refit by Dickson & Millero (1987) and KSO_4_ using (Dickson, 1990). The calculations for these parameters also included temperature, salinity, silicate and phosphate data. pH and CO_2_ levels remained relatively constant throughout the 80 days exposure period for the control and stable treatments (Table 1). A_T_, temperature, salinity, silicate, and phosphate showed no differences between CO_2_ treatments.

### Sample preparation

Live and dead *L. glaciale* thalli from each treatment (live: *n* = 3, dead: *n* = 3) were sampled after 80 days exposure. Samples were selected at random from each mesocosm; within the control treatment three of the six mesocosms were chosen at random. Thalli were air dried, embedded in resin (Buehler EpoxyCure, Düsseldorf, Germany), transverse sectioned (Buehler Petrothin) and polished using Buehler graded silicon carbide papers.

### Physical molecular structure

Changes in the physical structure of the skeleton at the molecular level were detected using Raman spectroscopy. Raman spectroscopy uses laser light to determine the molecular vibrational modes of substances, providing information on crystallographic structure, composition and stability. Raman spectroscopy was conducted using a Renishaw inVia Raman equipped with a Leica DM 2500M (Leica Microsystems GmbH, Wetzlar, Germany) microscope using a 785 nm laser and 1200 l mm^−1^ grating within the School of Geographical and Earth Sciences at the University of Glasgow (Glasgow, UK). Peak presence or absence at 1014 cm^−1^ Raman shift was recorded. Frequency and full width half maximum (FWHM, the peak width at half the peak height) were calculated for the V_1_ lattice mode (peaks nominally at a Raman shift of ca. 1089 cm^−1^). Raman spectroscopy was conducted on the spring–time deposited portion of each growth band to ensure temperature-dependent Mg concentrations remained the same across treatments. The spring-time growth portion was identified from Alizarin red stained calibration individuals in parallel experiments (e.g., Kamenos *et al*., 2008). Peak parameters were compared using a general linear model (assumptions of normality and heterogeneity of variance were met).

#### Bicarbonate presence

The 1014 cm^−1^ peak in Raman spectra of high Mg-calcite indicates the presence of bicarbonate (HCO_3_^−^) within the analysed sample (Bischoff *et al*., 1985)

#### Carbonate ion positional disorder

Band width (FWHM) of the ca.1089 cm^−1^ Raman spectrum peak is positively related to Mg concentrations and most likely results from positional disorder of the carbonate ion (increasing rotation of CO_3_^2−^ out of the basal plane) (Bischoff *et al*., 1985).

#### Composition

The frequency of the ca. 1089 cm^−1^ peak in spectra of biogenic calcite is primarily controlled by the Mg content (Bischoff *et al*., 1985). As the Mg content increases, the peak moves from 1085 cm^−1^ in calcite containing <3.9 mol% MgCO_3_, to 1094 cm^−1^ in Magnesite (25 mol% MgCO_3_) (Bischoff *et al*., 1985, Urmos *et al*., 1991).

### Net calcification, photosynthesis and respiration

Calcification and respiration of *L. glaciale* were determined in control and low, stable pH treatments after 80 days exposure to experimental conditions. Data are not available for the low, abrupt pH treatment. Live thalli (*n *= 3 *per* treatment) were incubated in 170 ml chambers for ca. 20 min during the day (PAR: ca. 90 photons μmol m^−2^ s^−1^) and at night (>1 h after sunset). Dead thalli (*n* = 3 *per* treatment) were incubated in 170 ml chambers for ca. 10 h spanning both day and night. Water motion was maintained using magnetic stirrers. A_T_ and dissolved oxygen concentrations were determined at the start and end of each experimental incubation. Calcification was determined using the total alkalinity anomaly technique (Smith & Key, 1975). Oxygen consumption / production was determined following the technique of Martin et al. (2013) using an oxygen electrode (1302 Oxygen Electrode; Strathkelvin Instruments) attached to a calibrated oxygen meter (Oxygen Meter 781; Strathkelvin Instruments). Comparisons were conducted using one and two-way general linear models (assumptions of normality and heterogeneity of variance were met).

## Results

### Bicarbonate presence

Bicarbonate was not present in live *L. glaciale* coralline algal thalli cultured under control conditions, but was present in increasing proportions within live thalli cultured under stable low pH and abruptly changing pH conditions (Fig. 1). All dead thalli contained HCO_3_^−^ (Fig. 1).

### Carbonate ion positional disorder

**Figure 2:**
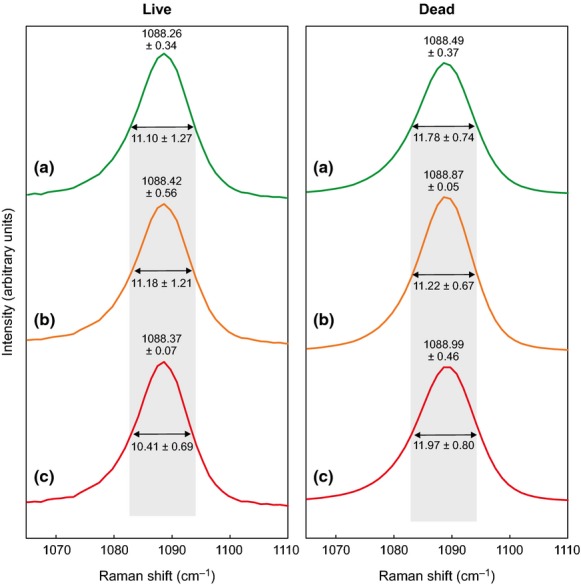
Nominal 1089 cm^−1^ peak Raman spectra characteristics. Unsmoothed full width half maximum (FWHM, double-headed arrow) and frequencies (above peak) of the ca. 1089 cm^−1^ peak in control (a), low, stable pH (b) and low, abrupt pH (c) treatments (*n* = 3 ± SD). Grey band indicates FWHM of the control treatments for comparison.

There were no significant differences in V_1_ lattice mode (symmetric stretch at ca. 1089 cm^−1^ frequency) between treatments within live (F_2, 14_ = 0.15, *P* = 0.865) or dead thalli (F_1, 14_ = 3.01, *P* = 0.105) (Fig. 2).

### Composition

In live coralline algae, we observed no significant change in peak frequency (Mg content) between individuals cultured in control, low pH and low, abruptly changed pH (F_2, 12_ = 1.28, *P* = 0.313) (Fig. 2). In the low, abrupt pH treatment, live coralline algae had a significantly reduced peak frequency (lower Mg content) compared to dead thalli in the same treatment (F_1, 12_ = 6.39, *P* = 0.026) (Fig. 2).

### Net calcification, dissolution, respiration and photosynthesis in live thalli

**Figure 3:**
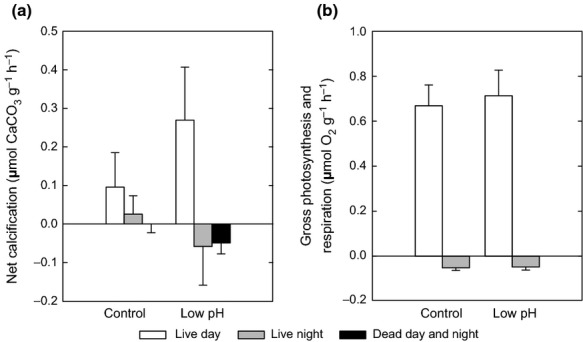
Algal calcification, dissolution and respiration. (a) Net calcification in the light (white) and dark (grey) in live and dead (black: light and dark) *Lithothamnion glaciale* under control and low, stable pH treatments, and (b) respiration and gross photosynthesis of live *L. glaciale* under control and low, stable pH treatments during the day (white) and night (grey). Data presented as mean ± SD.

*Lithothamnion glaciale* calcified during the day in both control and low, stable pH treatments (Fig. 3a). At night, *L. glaciale* calcified in the control treatment but dissolved in the low pH treatment (Fig. 3a). Under low, stable pH, *L. glaciale* calcified significantly more during the day than they dissolved at night, they also calcified marginally more in low pH than control treatments during the day (F_1, 54_ = 50.78, *P* < 0.001) (Fig. 3a) (calcification was not measured in low but abruptly changing pH). There were no significant differences in the photosynthesis (F_1, 22_ = 1.33, *P* = 0.261) or respiration (F_1, 24_ = 0.60, *P* = 0.446) of *L. glaciale* between the control and low, stable pH treatments (Fig. 3b)

### Dissolution in dead thalli

Dead thalli in low, stable pH conditions dissolved faster than control thalli (Fig. 3a) (F_2, 42_ = 4.59, *P* = 0.016). However, dissolution of dead thalli over the whole diel period was not significantly different from the dissolution of live thalli at night (F_1, 24_ = 0.13, *P* = 0.721) in the low, stable pH treatment (Fig. 3a).

## Discussion

We observed rate-dependent pH impacts on the physical molecular-level structure of red coralline algae (*L. glaciale*) after exposing thalli to low pH, low but abruptly reduced pH, and control conditions for 80 days.

### Bicarbonate presence

All dead thalli contained HCO_3_^−^, indicating that dissolution took place even under control pH conditions (Fig. 1). We suggest that the presence of HCO_3_^−^ indicates a chemical breakdown of the thallus skeletal material, possibly indicating the first stages of major physical breakdown. The higher percentage of individuals containing HCO_3_^−^ (Fig. 1) in the thalli exposed to the abrupt change in pH indicates that faster rates of change increase dissolution. The observed HCO_3_^−^ is unlikely to be from an external source as it was present in dead, but not live, thalli from the control treatment; an external source would have introduced the bicarbonate to both live and dead thalli.

As Raman spectroscopy was conducted on thallus calcite that was within each algal annual growth band, rather than calcite directly exposed to the sea water on the outer surface of the thallus, this suggests that damage was, at least, internal. In live algae this may be *via* (i) seawater ingress through low pH-damaged epithelium (Burdett *et al*., 2012), explaining the absence of damage in live thalli from the control treatment; or (ii) disruption of the calcification process at the centres of calcification.

### Carbonate ion positional disorder

While we observed no significant differences in V_1_ lattice mode (symmetric stretch at ca. 1089 cm^−1^ frequency) between treatments within live or dead thalli, we believe it is reasonable to attribute the modest fall of FWHM in live algae exposed to low, abrupt pH to a decrease in positional disorder (Fig. 2). Less positional disorder indicates longer, and thus weaker, Mg-O bond lengths (Bischoff *et al*., 1985). Thus despite the differences not being statistically significant, the longer, weaker Mg-O bonds indicate that live algae exposed to abrupt reductions in pH also show evidence of weakening at the molecular bonding level, an effect not observed in thalli exposed to control or low, stable pH treatments.

### Mg content

Absence of treatment induced differences in Mg content (peak frequency) suggests that, while alive, *L. glaciale* thalli were capable of buffering against the rate and magnitude of external carbonate chemistry changes, enabling them to continue to deposit high Mg calcite.

Reduced peak frequency of live over dead algae in the low, abrupt pH treatment indicates that live *L. glaciale* thalli were capable of lowering the Mg content of their calcite, making it less reactive to acidified conditions. A similar process may also occur in *Neogoniolithon* sp., another red coralline alga, which reduces the Mg/Ca ratio of the high Mg-calcite deposited at high *p*CO_2_ concentrations (>2000 μatm) (Ries, 2011). Thus, although epithelial damage may allow dissolution of the high Mg skeleton (Fig. 3), this does not appear to preferentially dissolve out Mg. This absence of Mg dissolution in the low, abrupt pH treatment could be due to the incorporation of Mg into the calcite crystal lattice (Kamenos *et al*., 2009) rather than being associated organic material.

### Calcification, dissolution, respiration and photosynthesis in live algae

When calcifying, *L. glaciale* appears to be able to compensate for OA-induced dissolution at night by increasing its calcification rate during the day. In fact, in low, stable pH conditions, *L. glaciale* up-regulated its calcification rates. Day-time (light) calcification rates in low pH were twice the rate required to maintain calcification in control conditions (Fig. 3). This suggests that, under OA conditions, *L. glaciale* may not only persist, but also increase their calcification, a phenomenon that has been observed in other algal and invertebrate species (Findlay *et al*., 2011, Martin *et al*., 2013, Rodolfo-Metalpa *et al*., 2011).

However, enhanced calcification in low, stable pH conditions was not supported by a change in photosynthesis (Fig. 3b), and thus an increase in available energy. While it was expected that coralline algae would obtain a photosynthetic benefit under OA (Ries *et al*., 2009), we observed no differences in photosynthesis or respiration of live *L. glaciale* between the control and low, stable pH treatments (Fig. 3b). Thus, live *L. glaciale* neither became metabolically challenged (which would have led to increased respiration), nor obtained photosynthetic benefits from changes in carbonate chemistry projected for 2100. We suggest that, during the day, energy usage required for enhanced calcification in thalli exposed to low, stable pH may limit photosynthetic efficiency, while at night the rate of dissolution may not be high enough to induce an increase in respiration. Over the whole diel period it is possible that the high energy requirement of day-time calcification in low pH conditions leads to energy reallocation away from night-time respiration. This may explain the absence of an up-regulation in respiration by individuals from the low, stable pH treatment. However, respiratory CO_2_ may not be exported as efficiently in low pH conditions, due to the higher CO_2_ concentration in the surrounding acidified water, potentially further enhancing night-time dissolution.

### Dissolution in dead algae

In dead thalli there is no protective epithelium and they cannot therefore buffer against changes in OA-associated carbonate chemistry, explaining their susceptibility to dissolution.

### Wider implications of coralline algal damage

Calcifying algae are particularly important for ecosystem function in both temperate and tropical ecosystems through their roles in (i) carbon cycling; (ii) provision of habitats and associated biodiversity hotspots, (iii) association with recruitment processes; and (iv) being major structural components of coral reef systems (Nelson, 2009). Thus, any change in the three dimensional structure and structural integrity of coralline algae may have significant effects on the ecosystem functions with which they are involved. For example live, structurally complex red coralline algae host high macro-organismal diversity (Biomaerl Team, 1999), and their ability to act as a nursery area is hierarchically controlled by the presence of a live epithelium and their complex three dimensional skeletal heterogeneity (Kamenos *et al*., 2004a). Thus, while we and others (e.g., Martin *et al*., 2013) show evidence of increased calcification under OA, this does not necessarily result in repair of damaged skeletons, or mean that the newly calcified skeleton is as physically strong as that deposited under higher pH conditions. Weaker structural integrity may make coralline algae more prone to fragmentation, thus impacting their role in ecosystem function; this will likely vary among species and geographic locations.

When producing historical climate records from red coralline algae there is a requirement that no material is lost from the algal thallus post-deposition (Burdett *et al*., 2011, Kamenos, 2010). Such loss, for example due to grazing, significantly lowers the resolution of the reconstructed record, as the temporal constraints of material loss is unknown. In particular, this makes constructing a time series chronology particularly difficult. Our results suggest that while red coralline algae are alive, OA is not likely to affect the records they lay down at >1 day resolution due to enhanced day-time calcification.

We show that the extent of damage caused by low pH conditions is dependent on the rate of change in carbonate chemistry and live / dead status in algal biomineralisers. This is of major concern as both live and dead coralline algae are critical in service provision due to their complex three-dimensional skeletal heterogeneity (Kamenos *et al*., 2004a) and coral reef stabilization / recruitment roles (Webster *et al*., 2013). In dead algae, the absence of post-dissolution repair mechanisms under projected OA conditions place the services they provide at significant risk. In addition, historical climate records held by dead algae within deposits are at risk from increased thallus dissolution, which is of particular concern as fossil deposits contain the longest palaeoclimatic records due to their age (Kamenos, 2010). It is thus likely that the ability of marine biomineralisers to cope with projected changes in marine carbonate chemistry and their ability to continue to provide services will be determined by the rate at which future *p*CO_2_ enrichment occurs.

## Supporting Information

**Table S1.** Table of nutrient concentrations within treatments.

## References

[b1] Agnew DJ, Taylor AC (1986). Effects of oxygen tension, temperature, salinity and humidity on the survival of two intertiday gammarid amphipods. Marine Ecology Progress Series.

[b2] Anthony KRN, Kleypas J, Gattuso J-P (2012). Coral reefs modify their seawater carbon chemistry - implications for impacts of ocean acidification. Global Change Biology.

[b3] Biomaerl Team (1999). Final Report (in 2 vols.), BIOMAERL Project.

[b4] Bischoff WD, Sharma SK, Mackenzie FT (1985). Carbonate ion disorder in sythnetic and biogenic magnesian calcites: a Raman spectral study. American Mineralogist.

[b5] Blackford J, Jones N, Proctor R, Holt J, Widdicombe S, Lowe D, Rees A (2009). An initial assessment of the potential environmental impact of CO2 escape from marine carbon capture and storage systems. Proceedings of the Institution of Mechanical Engineers Part a-Journal of Power and Energy.

[b6] Brewer PG, Riley JP (1965). The automatic determination of nitrate in sea water. Deep Sea Research.

[b7] Burdett HL, Aloisio E, Calosi P, Findlay HS, Widdicombe S, Hatton A, Kamenos NA (2012). The effect of chronic and acute low pH on the intracellular DMSP production and epithelial cell morphology of red coralline algae. Marine Biology Research.

[b8] Burdett HL, Kamenos NA, Law A (2011). Using coralline algae to understand historic marine cloud cover. Palaeogeography Palaeoclimatology Palaeoecology.

[b9] Caldeira K, Wickett ME (2003). Anthropogenic carbon and ocean pH. Nature.

[b10] Chave KE (1954). Aspects of biogeochemistry of magnesium, 1, Calcareous marine organisms. Journal of Geology.

[b11] Dickson AG (1990). Thermodynamics of the dissociation of boric acid in synthetic seawater from 273.15 to 318.15 K. Deep Sea Research.

[b12] Dickson AG, Millero FJ (1987). A comparison of the equilibrium constants for the dissociation of carbonic acid in seawater media. Deep Sea Research.

[b13] Dickson AG, Sabine CL, Christian JR (2007). Guide to best practices for ocean CO_2_ measurements. PICES Special Publication.

[b14] Duarte CM, Hendriks IE, Moore TS, Olsen YS, Steckbauer A, Ramajo L, Mcculloch MT (2013). Is Ocean Acidification an Open-Ocean Syndrome? Understanding Anthropogenic Impacts on Seawater pH.

[b15] Feely RA, Sabine CL, Hernandez-Ayon JM, Ianson D, Hales B (2008). Evidence for upwelling of corrosive “acidified” water onto the continental shelf. Science.

[b16] Findlay HS, Kendall MA, Spicer JI, Turley C, Widdicombe S (2008). Novel microcosm system for investigating the effects of elevated carbon dioxide and temperature on intertidal organisms. Aquatic Biology.

[b17] Findlay HS, Wood HL, Kendall MA, Spicer JI, Twitchett RJ, Widdicombe S (2011). Comparing the impact of high CO2 on calcium carbonate structures in different marine organisms. Marine Biology Research.

[b18] Foster MS (2001). Rhodoliths: between rocks and soft places. Journal of Phycology.

[b19] Grasshoff K (1976). Methods of seawater analysis.

[b20] Gruber N, Hauri C, Lachkar Z, Loher D, Frolicher TL, Plattner G-K (2012). Rapid progression of ocean acidification in the California current system. Science.

[b21] Hall-Spencer JM, Rofolfo-Metalpa R, Martin S, Ransome E, Fine M, Turner SM, Buia M-C (2008). Volcanic carbon dioxide vents show ecosystem effects of ocean acidification. Nature.

[b22] IPCC Core Writing Team Pachauri Rk & Reisinger a Eds (2007). Climate change 2007: synthesis report: Contribution of Working Groups I, II and III to the Fourth Assessment Report of the Intergovernmental Panel on Climate Change.

[b23] Irvine LM, Chamberlain YM (1994). Part 2B Corallinales. Hildenbrandiales.

[b24] Kamenos NA (2010). North Atlantic summers have warmed more than winters since 1353, and the response of marine zooplankton. Proceedings of the National Academy of Sciences of the United States of America.

[b25] Kamenos NA, Cusack M, Huthwelker T, Lagarde P, Scheibling RE (2009). Mg-lattice associations in red coralline algae. Geochimica et Cosmochimica Acta.

[b26] Kamenos NA, Cusack M, Moore PG (2008). Red coralline algae are global paleothermometers with bi-weekly resolution. Geochimica et Cosmochimica Acta.

[b27] Kamenos NA, Hoey T, Nienow P, Fallick AE, Claverie T (2012). Reconstructing Greenland Ice Sheet runoff using coralline algae. Geology.

[b28] Kamenos NA, Moore PG, Hall-Spencer JM (2004a). Attachment of the juvenile queen scallop [Aequipecten opercularis (L.)] to maerl in mesocosm conditions; juvenile habitat selection. Journal of Experimental Marine Biology and Ecology.

[b29] Kamenos NA, Moore PG, Hall-Spencer JM (2004b). The small-scale distribution of juvenile gadoids in shallow inshore waters; what role does maerl play ?. ICES Journal of Marine Science.

[b30] Kirkwood D (1989). Simultaneous Determination of Selected Nutrients In Seawater.

[b31] Kroeker KJ, Micheli F, Gambi MC (2012). Ocean acidification causes ecosystem shifts via altered species interactions. Nature Climate Change.

[b32] Kuffner IB, Andersson AJ, Jokiel PL, Rodgers KS, Mackenzie FT (2007). Decreased abundance of crustose coralline algae due to ocean acidification. Nature Geoscience.

[b33] Mantoura RFC, Woodward EMS (1983). Optimication of the indophenol blue method for the automated-determination of ammonia in esturaine waters. Estuarine Coastal and Shelf Science.

[b34] Martin S, Cohu S, Vignot C, Zimmerman G, Gattuso JP (2013). One-year experiment on the physiological response of the Mediterranean crustose coralline alga, Lithophyllum cabiochae, to elevated pCO2 and temperature. Ecology and Evolution.

[b35] Martin S, Gattuso JP (2009). Response of Mediterranean coralline algae to ocean acidification and elevated temperature. Global Change Biology.

[b36] Mehrbach C, Culberson CH, Hawley JE, Pytkowicz RM (1973). Measurement of the apparent dissociation constants of carbonic acid in seawater at atmospheric pressure. Limnology and Oceanography.

[b37] Melzner F, Gutowska MA, Langenbuch M, Dupont S, Lucassen M, Thorndyke M, Pörtner HO (2009). Physiological basis for high CO2 tolerance in marine ectothermic animals: pre-adaptation through lifestyle and ontogeny?. Biogeosciences.

[b38] Nelson WA (2009). Calcified macroalgae - critical to coastal ecosystems and vulnerable to change: a review. Marine and Freshwater Research.

[b39] Pierrot D, Lewis E, Wallace DWR (2006). MS Excel Program Developed for CO_2_.

[b40] Ragazzola F, Foster LC, Form A, Anderson PSL, Hansteen TH, Fietzke J (2012). Ocean acidification weakens the structural integrity of coralline algae. Global Change Biology.

[b41] Ries JB (2011). Skeletal mineralogy in a high-CO_2_ world. Journal of Experimental Marine Biology and Ecology.

[b42] Ries JB, Cohen AL, Mccorkle DC (2009). Marine calcifiers exhibit mixed responses to CO2-induced ocean acidification. Geology.

[b43] Rix LN, Burdett HL, Kamenos NA (2012). Irradiance-mediated dimethylsulphoniopropionate (DMSP) responses of red coralline algae. Estuarine Coastal and Shelf Science.

[b44] Rodolfo-Metalpa R, Houlbreque F, Tambutte E, Boisson F, Baggini C, Patti FP, Hall-Spencer JM (2011). Coral and mollusc resistance to ocean acidification adversely affected by warming. Nature Climate Change.

[b45] Smith SV, Key GS (1975). Carbon dioxide and metabolism in marine environments. Limnology and Oceanography.

[b46] Urmos J, Sharma SK, Mackenzie FT (1991). Characterization of some biogenic carbonates with Raman spectroscopy. American Mineralogist.

[b47] Webster NS, Uthicke S, Botte ES, Flores F, Negri AP (2013). Ocean acidification reduces induction of coral settlement by crustose coralline algae. Global Change Biology.

[b48] Williams B, Halfar J, Steneck RS, Wortmann UG, Hetzinger S, Adey W, Joachimski M (2011). Twentieth century delta C-13 variability in surface water dissolved inorganic carbon recorded by coralline algae in the northern North Pacific Ocean and the Bering Sea. Biogeosciences.

[b49] Zhang JZ, Chi J (2002). Automated analysis of nanomolar concentrations of phosphate in natural waters with liquid waveguide. Environmental Science & Technology.

