# Supplementary material for: Coralline algal structure is more sensitive to rate, rather than the magnitude, of ocean acidification
Source: Glob Chang Biol. 2013 Oct 8;19(12):3621–8. doi: 10.1111/gcb.12351 (PMC4285748; doi:10.1111/gcb.12351)
Supplement: Supplementary file 1 — Table S1. Table of nutrient concentrations within treatments. [file gcb0019-3621-sd1.docx]

**Supporting information**

**Supporting Table 1**. Nutrient concentrations (µM) at the end of the experimental period for nitrite, nitrite + nitrate, ammonium, silicate and phosphate for the three pH treatments. All nutrients were characterised by a general increase throughout the 80 d experiment. Data presented as mean ± SD.

|  | **Control** | **Low, stable pH** | **Low, abrupt pH** |
| --- | --- | --- | --- |
| **Nitrite** | 0.45 ±0.00 | 0.45 ±0.00 | 0.45 ±0.01 |
| **Nitrite+nitrate** | 31.57 ±0.12 | 31.20 ±0.15 | 31.38 ±0.18 |
| **Ammonium** | 0.32 ±0.03 | 0.28 ±0.05 | 0.27 ±0.08 |
| **Silicate** | 17.52 ±0.03 | 17.67 ±0.02 | 17.76 ±0.29 |
| **Phosphate** | 7.22 ±0.03 | 7.18 ±0.04 | 7.12 ±0.08 |
